# Coping with the hospital environment during the COVID-19 pandemic: A qualitative study of the survivors' perspective during their stay at the ICU and inpatient ward

**DOI:** 10.1016/j.heliyon.2024.e24661

**Published:** 2024-01-13

**Authors:** Manuel Herrero-Montes, Paula Parás-Bravo, Diego Ferrer-Pargada, César Fernández-de-las-Peñas, Luis Manuel Fernández-Cacho, Domingo Palacios-Ceña

**Affiliations:** a Departamento de Enfermería, Universidad de Cantabria. Instituto de Investigación Sanitaria Valdecilla (IDIVAL), Grupo de Investigación en Enfermería, Santander, Spain; b Servicio de Neumología, Hospital Universitario Marqués de Valdecilla, Santander, Spain; c Research Group of Manual Therapy, Dry Needling and Therapeutic Exercise (GITM-URJC), Department of Physical Therapy, Occupational Therapy, Physical Medicine and Rehabilitation, Universidad Rey Juan Carlos, Alcorcón, Spain; d Departamento de Enfermería, Universidad de Cantabria, Spain; e Research Group of Humanities and Qualitative Research in Health Science of Universidad Rey Juan Carlos (Hum&QRinHS), Department of Physical Therapy, Occupational Therapy, Physical Medicine and Rehabilitation, Universidad Rey Juan Carlos, Alcorcón, Spain

**Keywords:** MeSH): COVID-19, Hospital units, Intensive care units, Hospitals isolation, Pandemics, Patients, Hospital personnel, Qualitative research

## Abstract

The COVID-19 pandemic has significantly affected the clinical practice of healthcare professionals. This study aimed to explore the perspectives of COVID-19 survivors regarding the healthcare they received during their stay in the Intensive Care Unit (ICU) and the inpatient COVID-19 ward.

A qualitative case-study approach was implemented. Participants were recruited using non-probabilistic purposeful sampling strategy. Inclusion criteria included patients aged ≥18 years who received follow-up from the Pulmonology service at a Hospital in de North of Spain, were diagnosed with COVID-19 and bilateral pneumonia, and were admitted to the ICU before being transferred to a COVID-19 inpatient ward. Data was collected through in-depth interviews and researchers' field notes, and thematic analysis was performed. Techniques such as credibility, transferability, dependability, and confirmability were employed to ensure the trustworthiness of the data.

A total of 25 individuals (six women) were included in the study. Three main themes emerged from the analysis: common challenges faced in both units, coping with the hospital stay, and developing strategies. Findings highlighted the need to improve information dissemination, individualize care, and enhance direct patient interaction. Moreover, the study shed light on the psychological impact of hospitalization and ICU experience, including feelings of loneliness, confinement, and the lack of memories from the ICU stay, as well as the influence of care and healthcare language. Finally, strategies such as keeping the mind occupied and maintaining self-discipline were identified as crucial during hospitalization. These findings provide valuable insights for healthcare professionals in delivering care to individuals with COVID-19 in the ICU and hospital ward settings.

## 1 Introduction

As of fall 2022, more than 600 million cases of COVID-19 have been reported worldwide [1]. New symptoms are discovered daily, however, respiratory symptoms are the most characteristic and the most severe. The criteria for admission to an intensive care unit (ICU) includes the presence of clinical signs of pneumonia (depending on the severity and presence of risk factors) [2,3]. Patients with SARS-CoV-2 pneumonia have a higher in-hospital mortality compared to those with bacteraemic pneumococcal community-acquired pneumonia (B-PCAP). Patients requiring admission to the ICU for pneumonia are more likely to receive invasive mechanical ventilation (IMV) and have a higher mortality rate [4].

Hospitalization has implications for patient's emotional and psychological well-being, especially for those who are hospitalized in isolated rooms (inpatient wards) or in the ICU [5]. Patients admitted to the ICU with SARS-CoV-2 and in closed inpatient wards dedicated to patients infected with the virus, suffered conditions of extreme isolation, especially in the first months of the pandemic and in the first waves of COVID-19 transmission [6,7]. To minimize the impact of isolation, new ways of conveying information to patients, and their families, were developed using tablets and video calls [8,9]. However, despite the efforts of health care workers, isolation and fear hindered the humanization of care for patients admitted to the ICU [10]. This lack of humanization was characterized by minimal contact, the use of individual protective equipment that hindered communication and the ability for patients to identify staff, the lack of proximity or the impossibility of providing quality care to the professionals, among others [10].

An in-depth examination of the perspective of SAR-CoV-2 patients admitted to the different hospital units and departments at the beginning of the pandemic (first wave) is required. Furthermore, it remains imperative to persist in elucidating the constituents of healthcare as perceived by individuals themselves, across various clinical contexts [11,12], as well as the overall performance of professionals in the care of individuals with COVID-19, in order to produce a genuine and long-lasting shift in the current model of care [[13], [14], [15], [16]].

Qualitative research proves valuable in delineating intricate phenomena and comprehending the beliefs, values, and motivations that underpin the behaviors pertaining to individual health [17,18]. The experiences and expectations of COVID-19 survivors before and after treatment have also been investigated using qualitative investigations [19,20] and the experience of patients hospitalized for different pathologies has also been studied [[21], [22], [23]]. Answers to the following questions are necessary: What is the meaning of the hospital environment for individuals with COVID-19? What is their perspective during their admission and stay in the ICU and COVID-19 ward? What elements do they consider relevant or critical regarding the health service and professionals for their recovery? This study sought to describe the perspectives of COVID-19 survivors regarding the health care received during their stay in the ICU and in the inpatient COVID-19 ward.

## 2 Materials and methods

### 2.1 Study design

A qualitative descriptive case study with embedded units was conducted [24,25]. A case study can be composed of various units that aid in describing a phenomenon. These units could consist of various individuals from various contexts and locations who are only associated with the phenomenon being studied [24,25]. In this study, the phenomenon under study is the health care provided to patients with COVID-19 during their stay in the ICU and in inpatient units dedicated to patients infected with COVID-19. The Standards for Reporting Qualitative Research (SRQR) [26] and the Consolidated Criteria for Reporting Qualitative Research (COREQ) [27] were followed.

### 2.2 Ethics

The current study was approved by the Ethical Committee of Cantabria (code: 2020.416). This study was conducted in accordance with the Ethical Principles of the World Medical Association (Declaration of Helsinki). All participants gave their written consent prior to their inclusion in the study.

### 2.3 Research team

Five researchers (one woman) participated in this study, including one physiotherapist (CFP), three nurses (MHM, PPB, DPC) and one medical doctor (DFP). Each researcher had prior expertise conducting health sciences research. The researchers were strangers to one another and had no clinical connection to any of the individuals. The theoretical framework for this qualitative investigation, the researchers' viewpoints, and their reasons for doing the study were all defined prior to the study [26].

#### 2.3.1 Context

The present study was conducted during the first wave of COVID‐19 (March 14 to June 21, 2020). In Spain, the lockdown was activated at the national level, restricting the movements of all citizens [28]. Guidelines for infection prevention and control have been widely implemented as a result of the COVID-19 broad transmission. Personal protective equipment, segregating patients with respiratory illnesses from others, more stringent cleaning procedures, access control, and isolation wards are some of the tactics recommended in these guidelines [6,7].

In Spain, the criterion for hospital admission was the presence of clinical signs of pneumonia (depending on severity and the presence of risk factors) [2,3]. The patients remained in hospital wards or the ICU exclusively for COVID-19 patients, with isolation in single rooms. Direct face-to-face contact with relatives did not exist until the patient was discharged from the hospital.

### 2.4 Participants, and sampling strategies

In the present study, a non-probabilistic, purposeful sampling strategy was used, based on relevance to the research question rather than representativeness [29]. A purposeful sampling strategy involved deliberately selecting participants [29].

The inclusion criteria consisted of: a) Patients ≥18 years old being followed-up with the Pulmonology service of the Hospital Universitario de Valdecilla (HUV), b) diagnosed of COVID-19 disease through reverse transcription polymerase chain reaction (RT-PCR), c) who had been admitted to the ICU and were subsequently transferred to a COVID-19 inpatient unit. The exclusion criteria consisted of: a) individuals with COVID-19 who were not in the ICU and or COVID-19 inpatient ward, who did not have respiratory involvement, and/or with alterations in verbal communication.

Participants were recruited from the COVID-19 unit of the HUV Pulmonology Service. The specialist physician (pulmonologist) recruited the patients and then explained the study. If they accepted, another day was scheduled for data collection.

In qualitative research, a wide variety of proposals exist for justifying and determining sample size [[30], [31], [32]], also, there is no formula for the prior calculation of the sample size, since the results are not intended to be representative and generalizable [29]. In the current study, the sample size was determined following the proposal by Turner-Bowker et al. [33]. These authors reported that 99.3 % of concepts, themes, and contents emerged with 25 interviews [33]. With this proposal, a greater capacity to identify codes, categories, and topics is achieved. In addition, the current proposal also helps researchers to know when to stop collecting data and/or recruiting participants.

Thirty individuals who met the inclusion criteria were contacted and agreed to participate in the study. Of these, two withdrew from the study due to health problems, whereas three individuals stated that they did not feel psychologically prepared to be interviewed.

### 2.5 Data collection

In-depth interviews and researcher's field notes were used for data collection [29]. Data collection consisted of semi structured interviews that were based on a question guide designed to gather information regarding specific topics of interest (Table 1) [29]. The interviews used an open question such as: “Please, can you share your perspective regarding the care and health attention during your admission and stay in the ICU and COVID-19 ward?” In order to get the in-depth descriptions, open-ended follow-up questions were also used. The researchers took note of the themes and keywords found in the participants' answers and used their responses to elicit clarification from the participants about the content [29]. During the interviews, researchers used prompts to encourage participants to contribute more information by asking them to elaborate and clarifying what they had just said. This made it possible to gather pertinent information from the participants' points of view.

**Table 1:** Semi-structured interview guide.

Research areas	Questions
Diagnosis and hospital admission	Could you describe the process of COVID-19 diagnosis, what triggered you to seek help at the hospital, and what was the most relevant aspect of the hospital admission process for you?
Hospital stay	What was the most relevant aspect of your stay in the ICU? What was most relevant to you regarding your stay in the inpatient ward? How was the transition between the ICU and the inpatient ward? What was most relevant to you?
Treatment and recovery	What was your experience with the care and treatment you received? How was your relationship with the professionals? What was the most relevant aspect of your contact and relationship with them? How do you feel regarding the treatment received in the ICU and its outcome? And in the inpatient ward? What would you highlight as the most relevant aspect for you regarding your stay in the ICU? And in the inpatient ward?

^a^Intensive care unit.

The interviews were conducted by telephone. The interviews were audio-recorded and transcribed verbatim. Overall, 472 min of data collection were recorded, with a mean of 18.88 min (SD 7.82). The interviews were conducted by M.H-M. Also, researcher field notes were used as a secondary source of information to provide more in-depth information [29].

### 2.6 Data analysis

A thematic, inductive analysis was performed [29]. This kind of analysis, which takes into account the various viewpoints of the case study participants, is consistent with the design [25,34]. Complete verbatim transcriptions were drafted for each of the interviews, and researchers' field notes [29]. The thematic analysis [29] consisted of identifying the most descriptive content in order to obtain meaningful units, and subsequently reduce and identify the most common meaningful groups. In this manner, groups of meaningful units were formed, i.e., similar points or content that allowed the emergence of the topics that described the study participants’ experience [29]. This thematic analysis process was performed separately upon the interviews (by M.H-M, and D.P–C). Subsequently, joint meetings were held to combine the results of the analysis. Also, the data collection and analysis procedures were discussed during these meetings. In the case of differences in opinion, theme identification was performed based on consensus among the research team members. Subsequently, the research team held joint meetings to show, combine, integrate, and identify final themes [35]. No data analysis software was used.

### 2.7 Rigor

The techniques performed and application procedures used to control trustworthiness are described in Table 2 [36].

**Table 2:** Trustworthiness criteria.

Criteria	Techniques Performed and Application Procedures
Credibility	Investigator triangulation: each interview was analyzed by two researchers. Thereafter, team meetings were performed in which the analyses were compared, and themes were identified.
	Triangulation of data collection methods: in-depth semi-structured interviews were conducted, and researcher field notes were kept.
	Member checking: this consisted of asking the participants to confirm the data obtained during the data collection. All participants were offered the opportunity to review the audio records to confirm their experience. None of the participants made additional comments.
Transferability	In-depth descriptions of the study were performed, providing details of the characteristics of researchers, participants, contexts, sampling strategies, and the data collection and analysis procedures.
Dependability	Audit by an external researcher: an external researcher assessed the study research protocol, focusing on aspects concerning the methods applied and study design.
Confirmability	Investigator triangulation, data collection and analysis triangulation. Researcher reflexivity was encouraged via the completion of reflexive reports and by describing the rationale for the study.

## 3 Results

Twenty-five individuals (six female participants, 24 %) were recruited. The mean age of participants was 62.79 years (standard deviation, SD: 13.23). Table 3 details the demographic and clinical features of the sample.

**Table 3:** Demographic and clinical features of the sample.

Age	Mean: 62.79 SD: 13.23
Sex	Female, n = 6 Male, n = 19
Days in the ICU	Mean: 16.29 SD: 22.97
Days on the COVID-19 ward	Mean: 11.71 SD: 7.98
Mechanical ventilation	Yes, n = 15 No, n = 10
Oxygen therapy (type)	Nasal glasses, n = 3 Tnaf (high flow nasal therapy), n = 20 Venturi type mask, n = 2
Respiratory physiotherapy (ICU/Ward)	Yes, n = 21 No, n = 4

Three themes emerged from the data analyzed: a) Common challenges of both units; b) Facing the hospital stay, with two categories, Inpatient unit experience and ICU experience; and c) Developing strategies. Several participant narratives from the interviews that are directly related to the emerging themes have been presented in the description of each theme.

### 3.1 Common challenges of both units

Most patients recounted how during their stay on both units, they felt well cared for. The adjectives they used to define their care were, “wonderful,” “lovely,” “excellent,” and “phenomenal.” However, that did not mean that there was no room for improvement in care.

In both units, patients described how the information received by professionals was contradictory, some saying one thing and others the opposite in relation to their treatment and/or care. In addition, there was a lack of regular and continuous communication of information regarding evolution, many professionals were reluctant to inform patients, or sometimes the information given was vague and they did not clarify or inform them of anything, causing further uncertainty. In the words of one patient: *“What one [professional] said in the morning, in the afternoon it already changed. Nobody knew what was right. Neither did they.” (C06).*

Patients pointed out that there was no individualization of care, procedures were applied blindly, without asking the patient, with the justification that “the protocol was being followed".

The patients reported that there was a high turnover of staff within the units, meaning that they did not get to know all the people who cared for them. They perceived that there were many “young” people just out of college, with no experience, who asked questions rather than performed duties.

The patients were aware that they experienced delays due to the lack of staff, equipment, and the necessary isolation due to the virus, however, this did not justify some behaviors. For example, they pointed out that despite the high staff turnover, there was never enough staff, and there was a long delay in applying care and treatment, such as personal hygiene, mobilization, and care of devices. One patient recounted his experience with toileting: *“I had to wait an hour and a half for them to come and wash and clean me after I had soiled myself, as I couldn't hold it in, so I had to share an hour and a half of foulness, and the next day the same thing happened, and I had to call my family outside with my mobile phone, so they could call the nursing station and alert them to come to my room.” (C13)*

### 3.2 Coping with the hospital stay

#### 3.2.1 Experience in the inpatient unit

The patients described how they were not fully aware of everything until they arrived at the ward. Until then, they had difficulty remembering their admission to the ICU, only recalling specific moments and frozen images. Upon arriving to the inpatient ward, they began to understand everything that has happened to them and required answers and the presence of their loved ones.

The patients placed great emphasis on the feeling of loneliness they felt during their hospital stay. They could not interact with anyone, nor receive visitors. Many patients reported feeling like they had “the plague”, while others felt locked up, as if they were being punished at a cell in a concentration camp. This had a very strong emotional toil for them. One patient related: *“The six days I was there left me very marked, I felt lonely, mind you, without the family, I couldn't do anything, all* day *there, you know? But I was very lonely [Cries]." (C11).* Moreover, another patient noted: *“I was able to visit a concentration camp in Poland and see the punishment cells and, yes, it evokes a lot of that kind of thing in you."(C13).*

Patients reported that there were long periods of time when they remained alone and unaccompanied. Admittedly, family visits were forbidden during the first wave of the pandemic, however, no professional spent time with them, their visits were scarce and were limited to performing procedures, applying treatments or making assessments; few people invested time in being with them: *“She [the nurse] didn't see me physically, we talked on the phone, I asked her to come and see me, subtly, she said no, that right now she couldn't. I understood that, but you were left there, and you were alone.” (C14).*

Some patients justified this loneliness, because they themselves did not want to ask for help or were ashamed to tell the professionals, knowing the circumstances in which they were working during the pandemic (a large amount of patients, high hospital overload, lack of equipment, contagions, etc.). They did not want to bother or burden the professionals with their doubts or fears. Some waited until the symptoms worsened to call the professionals: *" … I felt like a fool for calling, even though I felt sick and had a fever. It seemed to me that [my symptoms] were being attended to poorly.”(C03)*

### 3.3 Experience on the intensive care unit

Most patients recounted how they did not have very clear memories of their stay at the ICU, many saying that they were hardly aware of anything during their stay. They related that they alternated episodes of consciousness and awakening with periods of “forgetfulness” and blackout and were unaware of what was going on. For some patients, being in the ICU was like a “mental black hole” in which they could not remember anything. One patient offered a description of this experience: *" … I remember eating with my family and feeling sick, blacking out and suddenly you open your eyes, you notice that you have a tube in your mouth coming out of a box and you see a brown liquid coming out, and hot, very hot. I started to move and move, and suddenly, plum, plash, asleep again, blackout.” (C14).*

As patients recovered, they increased their periods of “awakening” and became more aware of everything that was going on around them. Patients recalled how it was very aggressive for them to hear technical medical expressions, because many of them were related to severity, death or worsening of the other patients around them, such as intubation, prone, exitus, and cannulation. They highlighted “intubation” and “prone” as words that they feared and dreaded. Some patients described how being conscious and being told by a professional that if they got worse they would have to be intubated, rather than constituting a motivating incentive, was experienced as a threat that meant a poor prognosis and death: *“Some people think they're encouraging you to recover if they tell you things like < if you don't recover you will be intubated>. They don't realize it's not up to me … they should tell it to the bug [virus]. It was a full-blown threat.” (C05*).

It was hard for them to perceive how, next to them or nearby, another patient was getting worse, or dying. Upon recalling these memories, patients described the experience as “horrible”, “very hard”, “like agony”. One patient recalled: *“I don't even like to remember the experience, it was horrible, because of what I saw … the guy next to me could die or have a tracheotomy. You witnessed it all from your cubicle.” (C01).*

The patients recounted how being in the ICU was sometimes “claustrophobic,” enclosed in their glass cubicles. Often, they felt uncomfortable inside the ICU, due to the presence of continuous noises and lights, the sound of alarms going off, the cold, feelings of thirst and dry lips and mouth, the lack of privacy and being unable to sleep for days at a time. In addition, some patients pointed out that they felt they had needles all over their bodies, wanting to move or talk but were unable to do so, how they felt they were urinating on themselves without being able to avoid it, and how they felt the endotracheal tube in their eye or face and were unable to remove it.

Patients perceived when professionals tried to conceal things, “cover some things up” or try to downplay the importance of some data. For example, when vital signs or ventilation monitoring alarms started going off. One patient described: *“I heard alarms everywhere, all the time. It scared and upset me. If it's an alarm it means something is wrong, right? And they kept telling me not to worry. It didn't make sense, I have to worry, I'm sick and in the ICU.” (C05).*

### 3.4 Developing strategies

During their stay, the patients pointed out that the main strategy and priority was to keep their minds busy, “calm”, so that they were not aware of what was going on around them. This required being very disciplined, having a daily routine of occupations and activities, such as doing everything the professionals told you to do, walking and counting steps, reading on a scheduled basis, listening to the radio and/or music. It is noteworthy that many patients described self-discipline upon admission in such a dramatic context. One patient described how the patients were alone and had to deal with the situation: *“You need steadfast aplomb. From the first moment it's very important not to give in to the situation, you must be strong and navigate in that sea of loneliness and reach the port, because if you give in for 1 min you're lost”. (C13)*

In the case of the ICU, patient strategies were more passive. They were directly dependent on the professionals and their margin of autonomy was limited. Patients described how they tried to detach themselves from everything going on around them (alarms, interventions, machinery/crane noises, conversations of professionals about the health status of other patients, etc.), trying to sleep continuously so as not to hear anything.

## 4 Discussion

Our results show areas of improvement perceived by patients during their admission to the ICU and the inpatient ward. In addition, there were differences between the experiences at the ICU and in the inpatient ward. Finally, patients developed strategies to improve their adaptation on both units.

### 4.1 Common challenges of both units

The relationship between users and professionals during hospitalization is a key issue for the quality of service perceived by the patient [23]. In our study, patients expressed a very high satisfaction with the treatment received by professionals during their hospital stay, in line with other authors who found gratitude and satisfaction with the work of health care professionals [[37], [38], [39]]. In contrast, some patients reported a certain dissatisfaction with some aspects of their relationship with healthcare professionals, such as poor communication with professionals, high staff turnover or scarcity of resources, similar results to those reported by other research on hospitalized COVID-19 patients [39,40]. Fernández-Castillo et al. [10] described how ICU nurses themselves expressed difficulty providing humanized care in these circumstances. Some examples were how the use of individual protective equipment such as masks and suits, together with the limitation of physical contact with the professionals in care, posed a barrier to communication [10].

Nevertheless, an attempt was made to solve or mitigate the problem by labeling personal protective equipment with the name and photographs of the healthcare personnel to facilitate patient identification and humanize care.

Indeed, some of the patients in our sample showed insecurity and distrust in the care provided by healthcare professionals, perceiving that there were constant changes in treatment protocols.

### 4.2 Coping with the hospital stay

The feeling of loneliness is a frequent sentiment in our participants. Previous studies [37,39] found that isolation prevented contact with family members and caused discomfort among patients. In contrast, hospitalization and separation from family members appears as a relief for other patients, because they felt it was a mechanism to reduce the risk of infecting others [39]. Another key aspect is the limited time the professionals spent with the patients in their room. This could be related to the professionals' fear of contagion, the overload of work during the pandemic and the scarcity of protective equipment [40]. The combination of isolation plus limited contact with professionals could lead to a great emotional toil, which was devastating during the hospitalization of patients during COVID-19.

Patients became aware of their situation in the last days of ICU admission or upon transfer to the inpatient unit. Our results show that patients begin to remember critical situations they have experienced, such as the death of other patients next to them, alarms and emergency situations, etc. Many of our participants reported a distorted perception and discomfort due to invasive techniques (venipuncture, endotracheal intubation, etc.). Post-ICU syndrome is a term coined in 2010 by the Society of Critical Care Medicine that causes physical, cognitive, and mental dysfunction after ICU hospitalization and has been widely described in COVID-19 patients [41]. Studies have shown that patients present fatigue, cognitive dysfunction (mental fog, memory problems, attention disorders), sleep disorders and even anxiety and depression, and their prevalence increases over time [41]. Programs that include strategies to improve discharge education for patients and families or communication between patients and healthcare professionals can improve the experience during this period [42].

The close accompaniment of family members during hospitalization was perceived as important by patients, but also professionals [43]. Contact with relatives, also via telematic means, is important for patients [8,9,37,39]. It seems important to reduce isolation in a single room to the time strictly necessary and to articulate interventions, such as video calls, that allow and facilitate communication between patients and their loved ones. In addition, providing help to older people who present difficulties in handling devices and offering psychological support could be two useful measures to improve the experience of the isolated patient.

### 4.3 Developing strategies

Patients tried to keep their minds occupied in an attempt to isolate themselves from their surroundings, and to establish routines such as exercise, and listening to the radio and/or music. Previous studies [37,38], point out that the development of physical activity routines adapted to the hospital environment, mental health therapies or conversation with professionals are important tools for patients to cope with their situation.

Another key aspect is rest and sleep. In the case of admission to the ICU, this was hampered by the noise generated by the staff's professional activity. Recorded music has shown an improvement in the quality of sleep-in hospitalized patients, both in critical care and conventional hospitalization [44].

## 5 Limitations

This study has limitations concerning generalizability. Additionally, variations in disease experiences may be explained by COVID-19 evolution and subtypes. A more thorough investigation of the effects of COVID-19 during patient admission and hospital stays must also take the opinions of the health professionals into account.

## 6 Conclusions

People who have suffered COVID-19 positively value health care during their stay at the ICU and inpatient ward. However, our results highlight the importance of recognizing different areas for improvement in the care of patients with COVID-19 during their stay in the ICU and in the inpatient ward.

These results can help to improve and modify protocols of care in situations of isolation of patients in ICUs and inpatient wards, and to drive changes in the internal organization of human resources and application of procedures (treatments and care). Further research is necessary to describe the perspective and the process of applying care and treatment by health professionals during the COVID-19 pandemic.

## Data availability statement

Data that support the findings of this study are available from the corresponding author upon reasonable request. The data are not publicly available due to privacy and ethical restrictions.

## Funders statement

The project was supported by a grant of Comunidad de Madrid y la Unión Europea, a través del Fondo Europeo de Desarrollo Regional (FEDER), Recursos REACT-UE del Programa Operativo de Madrid 2014–2020, financiado como parte de la respuesta de la Unión a la pandemia de COVID-19 (LONG-COVID-EXP-CM), by a grant from Next-Val 2021 de la Fundación Instituto de Investigación Marqués de Valdecilla (IDIVAL), and by a grant from the Novo Nordisk Foundation 0067235. The sponsors had no role in the design, collection, management, analysis, or interpretation of the data, draft, review, or approval of the manuscript or its content. The authors were responsible for the decision to submit the manuscript for publication, and the sponsor did not participate in this decision.

## CRediT authorship contribution statement

**Manuel Herrero-Montes:** Conceptualization, Data curation, Formal analysis, Funding acquisition, Investigation, Methodology, Resources, Validation, Writing – original draft, Writing – review & editing, Project administration, Software, Supervision, Visualization. **Paula Parás-Bravo:** Conceptualization, Data curation, Formal analysis, Funding acquisition, Investigation, Methodology, Project administration, Resources, Software, Supervision, Validation, Visualization, Writing – original draft, Writing – review & editing. **Diego Ferrer-Pargada:** Data curation, Funding acquisition, Investigation, Methodology, Resources, Software, Writing – original draft, Writing – review & editing. **César Fernández-de-las-Peñas:** Conceptualization, Data curation, Formal analysis, Methodology, Project administration, Supervision, Validation, Writing – original draft, Writing – review & editing. **Luis Manuel Fernández-Cacho:** Conceptualization, Data curation, Formal analysis, Funding acquisition, Software, Writing – original draft, Writing – review & editing. **Domingo Palacios-Ceña:** Conceptualization, Data curation, Formal analysis, Funding acquisition, Investigation, Methodology, Project administration, Resources, Software, Supervision, Validation, Visualization, Writing – original draft, Writing – review & editing.

## Declaration of competing interest

The authors declare that they have no known competing financial interests or personal relationships that could have appeared to influence the work reported in this paper.
